# 

**Published:** 1854-06

**Authors:** 


### CONTENTS.

ORIGINAL COMMUNICATIONS:— PAGE

ART. I.—Illinois State Medical Society, . . . . . . . 241
ART. II.—Report of Dr. N. S. Davis, M.D., . . . . . . 251

SELECTIONS:—

Hysteria, . . . . . . . . . . . . . 261

BOOK NOTICES:

Observations on the Cause, Nature, and Treatment, etc., . . . . 274
Lectures on the diseases of Infancy and Childhood, . . . . 276
Pneumonia. . . . . . . . . . . . . 277

EDITORIAL:—

Personal, . . . . . . . . . . . . . 279
The Seventh Aniversary of the American Medical Association, . . . 280
Semi-Annual Meeting of the Esculapian Society, . . . . . 283
Thirteenth District Medical Society, . . . . . . . . 285
Progress and Character of Cholera, . . . . . . . 286
Editorial Change, . . . . . . . . . . . 288

